# Vitamin D Levels and Depressive Symptoms during Pregnancy: A Prospective Pregnancy Cohort Study

**DOI:** 10.1155/2024/1788167

**Published:** 2024-08-08

**Authors:** Vilja Seppälä, Soile Tuovinen, Marius Lahti-Pulkkinen, Polina Girchenko, Sture Andersson, Katri Räikkönen, Kati Heinonen

**Affiliations:** ^1^Welfare Sciences, Faculty of Social Sciences, Tampere University, Tampere, Finland; ^2^Department of Psychology and Logopedics, Faculty of Medicine, University of Helsinki, Helsinki, Finland; ^3^Centre for Cardiovascular Science, Queen's Medical Research Institute, University of Edinburgh, Edinburgh, UK; ^4^Finnish National Institute for Health and Welfare, Helsinki, Finland; ^5^Research Unit of Clinical Medicine, Faculty of Medicine, University of Oulu, Oulu, Finland; ^6^Children's Hospital, Pediatric Research Center, University of Helsinki and Helsinki University Hospital, Helsinki, Finland

## Abstract

**Objective:**

Depressive symptoms during pregnancy increase the risk for adverse outcomes in women and children. Lower vitamin D levels are suggested to be associated with higher depressive symptoms in nonpregnant populations. We studied if the level of or change in serum of 25-hydroxyvitamin D (25(OH)D) concentration was associated with the levels of depressive symptoms during pregnancy.

**Methods:**

The participants of this prospective longitudinal study came from the Prediction and Prevention of Preeclampsia and Intrauterine Growth Restriction (PREDO) study. The analytic sample comprised 307 women (mean age = 32.5 (range 20.3–44.3)) who reported depressive symptoms concurrently with serum 25(OH)D measurements at a median of 13.0, 19.3, and 27.0 gestational weeks. Depressive symptoms were assessed using the Center for Epidemiologic Studies Depression Scale. Linear and mixed-model regression analyses were used to study the associations.

**Results:**

The 25(OH)D levels were not associated with depressive symptoms cross-sectionally (*p* values > 0.58) or across the three assessment points during pregnancy (*B* = −0.05; 95% CI, −0.12, 0.01; and *p* = 0.12). Yet, a higher increase in 25(OH)D during pregnancy was associated with lower levels of depressive symptoms (*B* = −1.41; 95% CI, −2.75, −0.07; and *p* = 0.04) but not after adjusting for covariates (*p* = 0.08).

**Conclusions:**

The 25(OH)D levels and depressive symptoms were not associated among pregnant women throughout the pregnancy. However, there is a need for randomized controlled trials to fully exclude the possibility of vitamin D supplementation in the prevention of depression during pregnancy.

## 1. Introduction

Depression is a major health problem among pregnant women: approximately 20% experience clinically relevant depressive symptoms, and over 10% suffer from depressive disorders [1, 2, 3, 4]. Prenatal depression is the biggest risk factor for postnatal depression [5], and in 40%–50% of cases, the symptoms continue postpartum [1, 6]. Depression during pregnancy also increases the risk for gestational diabetes, preeclampsia, and Caesarean section delivery and is associated with unfavorable outcomes in the children including low birth weight [7], preterm birth [7, 8, 9], and neurocognitive and mental health adversities [1, 3, 10, 11]. Hence, there is an urgent need to identify potentially modifiable factors associated with depression during pregnancy as they may serve as targets for novel preventive interventions.

One such modifiable candidate is maternal vitamin D status, which is clinically measured as serum 25-hydroxyvitamin D (25(OH)D) level, with a concentration <50 nmol/L considered vitamin D deficiency [12]. Vitamin D is a steroid hormone with a multifaceted function, including direct effects on brain development and function [13, 14, 15]. Vitamin D receptors have been identified in different parts of the brain [15, 16], and low vitamin D levels have been implicated as the link to neuropathology [13, 16]. Vitamin D has also an anti-inflammatory function [17], and inflammation during pregnancy has been associated with depression [18]. Already in early pregnancy, maternal metabolism of vitamin D changes drastically [19], and according to recent studies, 25(OH)D levels tend to increase [20, 21]. The fetus cannot synthesize its own vitamin D but relies on the placenta to transfer 25(OH)D from maternal bloodstream [19, 22]. A recent study showed that the placenta is not passively diffusing 25(OH)D to the fetal side but is actively taking up and breaking down 25(OH)D [22]. This study also demonstrated that maternal vitamin D levels induced changes in the placenta functionality as implicated by alterations in the expression of genes and proteins involved in cellular pathways, which are critical for the placenta's role in pregnancy, and by altering the placental epigenetic landscape [22]. Hence, in addition to the possibility that vitamin D is a biologically plausible link to maternal depression, it may also provide insight into the mechanisms connecting maternal depression to child outcomes.

Meta-analyses and reviews provide increasing evidence for an association between low 25(OH)D levels and depression in nonpregnant populations [23]. Similar associations have been suggested to exist among pregnant women [24, 25, 26, 27]. However, a recent systematic review concluded that the current evidence remains inconclusive due to the poor quality and heterogeneity of the studies [28]. There are methodological limitations including small sample sizes and restriction of the samples either to women likely to have depression or vitamin D insufficiency. The existing studies have also mainly measured vitamin D status and depressive symptoms only once during early pregnancy [28, 29, 30, 31, 32, 33, 34], thus limiting the conclusions of sensitive periods of 25(OH)D levels and leaving the results during second and third trimester even more contradicting [33, 34, 35]. However, there are four longitudinal studies using more than one measurement point of depressive symptoms [32, 33, 34]. All studies are relatively small (in all <180 participants) and focusing on populations with high prevalence of vitamin D insufficiency [29, 35] and/or to women likely to have high [34] or low [35] risk for depression, so the need for additional studies in differing populations remains. In the longitudinal studies, low early-pregnancy 25(OH)D levels have been reported to associate with higher depressive symptoms at third trimester [32, 34] and with increased risk of depressive symptoms throughout pregnancy [33]. Late pregnancy 25(OH)D levels or change across the third trimester were not associated with levels [34, 35] or changes in depressive symptoms [35]. Especially, the vitamin D levels during the second trimester and the change of vitamin D across pregnancy in relation to depressive symptoms remain mainly unstudied.

To address the limitations in the literature, in the current study, we tested whether higher serum 25(OH)D levels, measured at three times during pregnancy at mean of 13.0, 19.3, and 27.0 weeks of gestation, among 307 women, were associated with lower levels of depressive symptoms assessed concurrently with the 25(OH)D concentrations. We also studied whether change in the 25(OH)D levels across gestation was associated with levels of depressive symptoms during pregnancy. Finally, we conducted sensitivity analyses by excluding women with a history of depression diagnoses to rule out the potential bias due to the more severe cases.

## 2. Methods

### 2.1. Participants

The participants came from the Prediction and Prevention of Preeclampsia and Intrauterine Growth Restriction (PREDO) study. The PREDO study cohort was established to identify novel risk factors and biomarkers in pregnant women associated with the development of preeclampsia and intrauterine growth restriction and to study their associations with child developmental and health outcomes. Recruitment took place at 10 study hospitals in Southern/Eastern Finland when the women visited the first fetal ultrasound screening in early pregnancy. The PREDO study enrolled altogether 1,079 Finnish-speaking pregnant women between 2005 and 2009 with a known risk factor status for preeclampsia and intrauterine growth restriction (IUGR) who gave birth to a singleton live child. Of them, 969 had one or more, and 110 had none of the known risk factors for preeclampsia and IUGR. Due to economic constraints, blood was sampled only at the three largest study hospitals, and 425 women underwent venous blood sampling for 25(OH)D assessment at one to three time points during pregnancy. The study is described in detail elsewhere [36].

All participating women signed informed consent forms. The PREDO study protocol was approved by the Ethics Committees of the Helsinki and Uusimaa Hospital District (485/E7/2004, 1/2005). Furthermore, strengthening the Reporting of Observational Studies in Epidemiology (STROBE) statement was employed to report information of this study [37].

### 2.2. The 25-Hydroxyvitamin D Concentration

Blood was sampled from antecubital vein in the morning between 7 and 9 AM, after participants had fasted for at least 10 hr. Plasma was separated immediately, and samples were stored at −80°C until analyzed. The 25(OH)D concentration was analyzed with an IDS-iSYS fully automated immunoassay system with chemiluminescense detection (Immunodiagnostic Systems Ltd., Bolton, UK). Intra- and interassay coefficients of variability in the sample were <5% and 7%.

### 2.3. Depressive Symptoms

Depressive symptoms were measured using the Center for Epidemiologic Studies Depression Scale (CES-D), which is a self-administered questionnaire [38]. The 20 CES-D items describe depressive symptoms during the past week and are rated from none (0) to all the time [3]. A sum score ranges from 0 to 60. The women completed the CES-D biweekly up to 14 times throughout pregnancy between 12 and 13 and 38–39 gestational weeks/delivery. This allowed us to identify the assessments that matched the closest to the three blood samplings for 25(OH)D. The CES-D is a well-established measure of depression, and it has been validated in pregnant women [3, 11, 39, 40, 41].

### 2.4. Covariates and Moderators

Covariates known to be associated with vitamin D levels and/or depression used in the current study were season when vitamin D was sampled (spring (March to May), summer (June to August), autumn (September to November), winter (December to February)) [42], maternal age at childbirth (<40 vs. ≥40 years) [43], smoking during pregnancy (no/quit/smoked throughout) [44, 45], early pregnancy body mass index (BMI) (normal weight (BMI < 25 kg/m^2^)/overweight (BMI = 25–29 kg/m^2^)/obesity (BMI ≥ 30 kg/m^2^)) [46], diagnosis of hypertensive disorders (normotension/chronic hypertension, preeclampsia, gestational hypertension, or unspecified hypertension in current pregnancy (International Classification of Diseases, Tenth-Revision (ICD-10): I10, 010−011 and O13−O16)) [47, 48], and diagnosis of diabetes disorders (no/type 1, type 2, or gestational diabetes (ICD-10: E08−E14 and O24)) [49, 50], maternal alcohol use during pregnancy (no/yes) [51], and education level (secondary or less/lower tertiary/upper tertiary) [52]. Covariates were self-reported; except data for diagnoses were derived from medical records and verified by a clinical jury. Self-reported early pregnancy BMI was also verified in first antenatal clinic visit.

Additionally, we used depression diagnoses (ICD-10, F32–F33 and F341 since 1996, and ICD-9, 2961, 2968A, and 3004A in 1987–1995) for sensitivity analyses. Diagnoses were retrieved from the Care Register for Healthcare (HILMO), comprising diagnoses of all hospitalizations in Finland since 1969 and outpatient visits in specialized medical care since 1998. No women had bipolar disorder in our sample.

### 2.5. Statistical Analyses

The primary data analytic tools were linear regression and linear mixed-effects regression, which were selected to account for both the individual measuring points and the nested structure of repeated measurement data. No missing values were allowed in any of the analysis. Applying linear regression, we first tested cross-sectional associations between 25(OH)D levels and depressive symptoms, and associations between 25(OH)D levels at the previous measurement point and depressive symptoms in subsequent assessment points. These analyses were repeated applying logistic regression with dichotomized depressive symptoms (clinically significant levels of depressive symptoms; CES-D ≥ 16 points [38, 53]) and 25(OH)D variable (vitamin D deficiency; 25(OH)D <50 nmol/L [12]). Linear mixed-effects regression tested whether the 25(OH)D levels at the three sampling points, which we treated as a time-varying within-person predictor, were associated with depressive symptoms at these same sampling points as the time-varying outcome. To test if a *change* in vitamin D levels across the pregnancy was associated with depressive symptoms, we first defined the estimate of the change in vitamin D as the univariable linear regression slope where 25(OH)D levels were predicted by gestational week (estimated for each person separately), and second, we tested whether the change in vitamin D across pregnancy varied according to the level of depressive symptoms.

In the mixed-effects regressions, we specified variance component covariance structure. Because 25(OH)D and depressive symptom distributions were skewed, we normalized them with square root transformations. To facilitate interpretation, we transformed all continuous variables to standard deviation (SD) units (for time-varying variables, we used the mean of the three data points during pregnancy and its SD to retain within-person variation).

In the linear, logistic, and mixed-effects regressions, we made adjustments for the season at the time of 25(OH)D measurement and covariates that were statistically significantly associated with depressive symptoms. Mixed-effects models also included the gestational week when blood was sampled as a within-person time-varying predictor. Sensitivity analysis excluded mothers with a history of depression diagnosis.

The statistical analyses were conducted using IBM SPSS Statistics for Windows, version 28.0 (IBM Corp., Armonk, NY, USA), and SAS, vrsion 9.4 (SAS Institute Inc., Cary, NC, USA).

## 3. Results

### 3.1. Characteristics

Of the 1,079 women enrolled in the study and 425 women who underwent at least one blood sampling during pregnancy, our analytic sample comprised 307 (mean age = 32.5 (range 20.3–44.3)) women who reported depressive symptoms concurrently with the three blood samplings during pregnancy. The women in the analytic sample were more often younger than 40 years, of normal weight, and with no history of depression diagnosis before pregnancy compared with women with missing values in some of the blood samples or depressive symptom reports (Table 1). Table 1 presents the characteristics of the 307 pregnant mothers included in our analyses. The participants came for blood sampling at median (interquartile range) 13.0 (12.6–13.4), 19.3 (19.0–19.7), and 27.0 (26.6–27.6) gestational weeks. The 25(OH)D levels and the depressive symptoms showed a high rank-order stability across pregnancy (all *r* > 0.48, *p*  < 0.001, for 25(OH)D, and all *r* > 0.54, *p*  < 0.001 for depressive symptoms). The number of those with clinically relevant level of depressive symptoms (CES-D ≥16 points) and vitamin D deficiency (25(OH)D <50 nmol/L) was 16 (5.2%), 9 (2.9%), and 8 (2.6%) at first, second, and third sampling point, respectively.

Table 2 shows the associations between the covariates and the variables of interest (i.e., depressive symptoms and 25(OH)D levels). Women older than 40 years, who used alcohol during pregnancy or who were obese in early pregnancy, had higher depressive symptoms scores. Normal weight and obese women also had lower 25(OH)D levels (Table 2).

### 3.2. 25(OH)D and Depressive Symptoms during Pregnancy

Table 3 shows that 25(OH)D levels at any measurement point were not cross-sectionally associated with depressive symptoms, and neither the 25(OH)D levels in previous measurement point did predict depressive symptoms at the subsequent assessment points.  Table 4 show that results remained similar also when depressive symptoms and vitamin 25(OH)D levels were analyzed as dichotomized to elucidate association among more severe cases. Adjusting for season of measurement, age, alcohol use, and BMI did not substantially change the results (all *p* values > 0.07).

**Figure 1:**
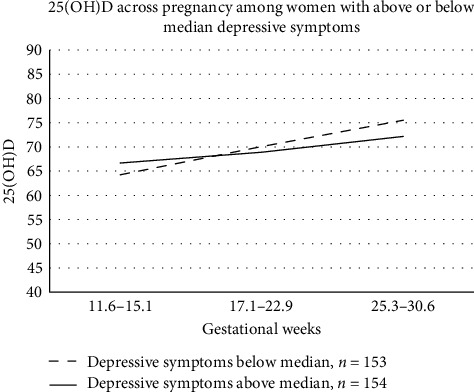
25(OH)D levels in women with depressive symptoms below and above the median across three measurement points during pregnancy. The change in 25(OH)D levels across pregnancy was negatively associated with depressive symptoms in linear mixed-model analysis (*B* = −1.41; 95% CI, −2.75, −0.07; and *p* = 0.039). In the figure, the change in 25(OH)D levels is shown with average values of 25(OH)D across the three measurement points.

Table 5 shows that 25(OH)D levels were not associated with depressive symptoms across the three assessment points in the linear mixed-effects regression analysis (*B* = −0.05; 95% CI, −0.12, 0.01; and *p* = 0.12, unadjusted model). However, a higher increase in 25(OH)D levels across pregnancy was associated with lower levels of depressive symptoms across pregnancy (*B* = −1.41; 95% CI, −2.75, −0.07; and *p* = 0.039, unadjusted model) (Table 5). This association attenuated after adjustment for covariates (*B* = −1.20; 95% CI, −2.54, 0.14; and *p* = 0.08). See Figure 1 for 25(OH)D levels in women with depressive symptoms below and above median across three measurement points during pregnancy. Excluding mothers with a history of depression diagnosis (*n* = 21) did not change the results (see Table 6).

## 4. Discussion

The aim of this study was to test if higher levels or increase in serum 25(OH)D were associated with lower levels of depressive symptoms across pregnancy. We found that 25(OH)D levels were not associated with self-reported depressive symptoms during pregnancy. However, we found that a higher increase in 25(OH)D levels was associated with lower levels of depressive symptoms across pregnancy. Nevertheless, the association attenuated after controlling for season, age, alcohol use, and BMI.

Lack of association between 25(OH)D levels and depressive symptoms during pregnancy found in our study is in line with the findings from meta-analysis [54] including 4,746 pregnant women from three cohort studies [28, 30, 33] and one intervention study [35]. In meta-analysis, vitamin D levels and depressive symptoms were reported one to three times during pregnancy in four different continents adding up to a comprehensive sample [54]. Among studies not included in the meta-analysis, one relatively large study (*n* = 498) reported a cross-sectional association between 25(OH)D and depressive symptoms measured at 15 weeks of gestation, which was rendered nonsignificant after controlling for season, gestational age at blood draw, BMI, smoking, race, education, leisure-time physical activity, and marital status [29]. Further, in line with the findings from our study, no association was also reported among women at high risk for depression at 34–36 weeks of gestation [34].

Our findings are inconsistent with the studies that have found an inverse association between 25(OH)D and depressive symptoms during pregnancy [24, 31, 32, 34]. Three studies, not included in the above-mentioned meta-analyses, have reported significant cross-sectional associations at the first/second trimester between 25(OH)D levels and depressive symptoms [31, 32, 34], and two of them have also reported longitudinal associations from first/second trimester 25(OH)D to third trimester depressive symptoms [32, 34]. These studies were limited by inclusion of only women at high risk for depression [34], small samples [32, 34], or inability to control important covariates (e.g., the season) [31, 32]; thus, these results are not fully comparable to ours.

Further, in the current study, 22.5%, 22.1%, and 24.1% of the participants had clinically relevant depressive symptoms (CES-D ≥16 points) at the three measuring points, respectively, which corresponds to other studies of validated depression scales reporting a prevalence of approximately 20% during pregnancy [2, 6, 55]. In some, although not in all [31, 32, 33], earlier studies, reporting significant associations between 25(OH)D and depressive symptoms during pregnancy, the prevalence of women with clinically significant levels of depressive symptoms has been higher, varying between 28% and 100% [28, 30, 34]. Further, the study, which excluded women with depressive symptoms over the clinical cut-point, did not find significant associations with 25(OH)D [35]. In our study, exclusion of the mothers with a history of depression diagnosis (*n* = 21) did not change the results. Moreover, in our study 16.6%, 15.3%, and 9.8% of the participants had vitamin D deficiency, i.e., 25(OH)D levels <50 nmol/L, respectively, at the three consecutive measurement points during pregnancy. In the studies reporting significant associations of 25(OH)D with depressive symptoms, the prevalence of deficient 25(OH)D levels has been often higher than in our study, varying between 21.6% and 82.6% [28, 30, 31, 32]. Similar deficiency levels to our study (at baseline 14.0%–16.2%) have been reported only in two studies [33, 34]. In our study, odds of clinically relevant depressive symptoms were not predicted by vitamin D deficiency indicating no association between depressive symptoms and 25(OH)D even in the high-risk population. However, our analyses of the association in high-risk population might not have been powered enough. Hence, it cannot be excluded that there would be an association between 25(OH)D and depressive symptoms in populations with elevated depressive symptoms or 25(OH)D deficiency or both. The suggestion of the association between depressive symptoms and 25(OH)D in high-risk populations is supported by the finding that in nonpregnant populations subjects with major depression and 25(OH)D deficiency particularly benefitted from vitamin D supplements [56].

At the same time, we found that higher increase in 25(OH)D levels was associated with lower levels of depression across pregnancy. However, the association attenuated after adjustment for season, age, alcohol use, and BMI. The only earlier study examining the change in 25(OH)D during pregnancy did not find a significant association with depressive symptoms [35]. In our study, the change was measured from early pregnancy to third trimester, whereas in the previous study, the changes in 25(OH)D levels were measured during the third trimester only [35]. Different periods of the changes in 25(OH)D measurements are one of the probable reasons for the discrepancy between the results. Our findings of increasing levels of 25(OH)D across the pregnancy are in line with recent studies [20, 21]. Increase of 25(OH)D levels across the pregnancy is reported also among women with no dietary supplementation of vitamin D [21], but the role of vitamin D supplementation in the observed increase of 25(OH)D levels in the current study cannot be excluded. The faster increase in the 25(OH)D level in mothers with less depressive symptoms in the current study might also be due to lifestyle behaviors. Lower depressive symptoms during pregnancy have been linked with healthier lifestyle behaviors, such as a healthy diet and outdoor leisure-time [57, 58]. These healthy behaviors are also likely to be associated with higher vitamin D levels [59]. Moreover, our finding that the association attenuated after adjusting for factors possibly reflecting lifestyle behaviors (i.e., alcohol use and BMI) supports this hypothesis. However, it is also possible that the faster increase in 25(OH)D levels during pregnancy buffers against depressive symptoms and that the association would be more evident in high-risk populations. Woman's body goes through a large amount of physical changes during pregnancy, including the changes in metabolism of vitamin D [19, 22, 60]. Moreover, the placenta has an active role in influencing both the mother's and fetus' levels of 25(OH)D and its metabolites [19, 22]. The role of these physiological changes in depressive symptoms is still largely unknown. However, it is important to note that the finding of the association between change of 25(OH)D and depressive symptoms across the pregnancy is novel, and the association attenuated after covariates were controlled for.

The strengths of our study include a prospective study design and state-of-art measurement of 25(OH)D throughout pregnancy. In our study, we had the possibility to control for the factors influencing vitamin D and depressive symptoms. We also had a relatively large sample size compared with previous longitudinal studies thereby enabling more reliable conclusions. One strength of our study is also that the variation of both depressive symptoms and 25(OH)D corresponds with the variation reported in pregnant populations in general [55, 61], enhancing the generalizability of the results. Although our sample characteristics are good for studying systematic association between depressive symptoms and 25(OH)D in pregnant population in general, in regard of high-risk populations with clinically relevant depressive symptoms and/or vitamin D deficiency, our sample size is small. Thus, the possibility of extending the conclusions for high-risk populations is limited. The lack of data on ethnicity is also a study limitation. The women participating in the study were all residing in Finland, while pregnant and future studies should examine whether similar findings emerge in other cultural or ethnic settings. Compared to attrition sample, women in our study were more often in normal weight class, and BMI is known to associate with vitamin D metabolization [46]. However, the women in our sample were still more often obese (27.4%) than pregnant women in Finland generally (11.5%) [62]. Women in our sample had less often history of depression diagnosis (7.2%) than women in attrition sample (12.0%) or women in Finland generally (10.9%–12.2%) [63, 64]. However, as stated above, there was no differences in depression symptom levels. It is possible that selection bias in participation may have influenced our findings. In our study, we used CES-D, which is not primarily designed for measuring depressive symptoms in pregnant women. However, CES-D has been validated in pregnant women [3, 11, 39, 40, 41], and it consists of very few items measuring depression criteria not fully applicable during pregnancy, such as weight gain or psychomotor agitation/retardation [38, 65]. Furthermore, in an earlier study of the PREDO sample, total CES-D scores and all its subscales during pregnancy have predicted postnatal depressive symptoms and well-being of the child [3]. In addition, we did not account for dietary supplementation of vitamin D. In Finland, pregnant women are recommended to use 10 *µ*g of vitamin D supplementation daily all year round, whereas for nonpregnant adult population, vitamin D supplementation is not systematically recommended [66]. Thus, we cannot exclude the effect of potentially increased supplementation use in the increase of 25(OH)D levels or in the association between serum 25(OH)D and depressive symptoms. However, although the specific impact of supplementation could not be evaluated, the used blood serum 25(OH)D is considered as a robust and reliable biomarker of general vitamin D status, covering different sources of vitamin D including food, sunlight exposure, and supplementation [67]. More general strengths and weaknesses of the PREDO study are comprehensively listed elsewhere [36].

## 5. Conclusions

In conclusion, our study suggests no association between 25(OH)D and depressive symptoms throughout the pregnancy. However, the potential association between 25(OH)D and depressive symptoms cannot be fully excluded as the current study is an observational study using a low-risk-of-vitamin-D deficiency population. There is thus a need for randomized controlled trials among high-risk pregnant women to rule out the possibility of vitamin D supplementation in the prevention of depression during pregnancy. In addition, in the unadjusted model, increasing levels of 25(OH)D were associated with depressive symptom across pregnancy thus warranting further studies on change. The importance to study the associations with the levels and change in 25(OH)D especially in pregnant populations comes from the knowledge that the metabolism of vitamin D changes during pregnancy [19] and from a need to find modifiable factors for therapeutical interventions of depressive symptoms.

## Figures and Tables

**Table 1:** Characteristics of the mothers who provided data on plasma 25(OH)D levels and depressive symptoms during pregnancy and those who did not provide either plasma 25(OH)D or depressive symptoms during pregnancy.

	Analytic sample total (*N* = 307), mean (SD) (*N* (%))	*N*	Attrition sample total (*N* = 772), mean (SD) (*N* (%))	*N*	Group difference (*p*)
Age (years), <40 years, *n* (%)	279 (90.9%)	307	623 (80.7%)	772	<0.001
Education, upper secondary or tertiary	164 (53.4%)	307	400 (54.1%)	740	0.85
Smoking during pregnancy, no	291 (95.1%)	306	734 (95.4%)	769	—
Alcohol use during pregnancy, no	260 (86.4%)	301	516 (81.5%)	633	0.06
Body mass index in early pregnancy (kg/m^2^)	—	307	—	772	0.002
Normal weight (<25 kg/m^2^)	166 (54.1%)	—	342 (44.3%)	—	—
Overweight (25–29.99 kg/m^2^)	57 (18.6%)	—	131 (17.0%)	—	—
Obese ((⩾30 kg/m^2^)	84 (27.4%)	—	299 (38.7%)	—	—
Hypertensive disorders in pregnancy, yes	99 (32.2%)	307	275 (35.6%)	772	0.29
Diabetes disorders in pregnancy, yes	63 (20.5%)	307	198 (25.6%)	772	0.08
History of depression diagnosis before pregnancy, yes	21 (7.2%)	291	76 (12.0%)	633	0.03
Depressive symptoms during pregnancy (mean of reports at three blood sampling points)	11.4 (7.4)	307	11.4 (7.6)	31	0.97
Depressive symptoms matching the blood sampling points					
1^st^ sampling point	11.5 (8.3)	307	11.2 (8.1)	29	—
2^nd^ sampling point	11.3 (8.6)	307	11.6 (8.2)	26	—
3^rd^ sampling point	11.4 (8.9)	307	11.0 (9.1)	28	—
Clinically relevant (≥16 points in CES-D) depressive symptoms matching the blood sampling points					
1^st^ sampling point	69 (22.5%)	307	9 (31.0%)	29	—
2^nd^ sampling point	68 (22.1%)	307	9 (34.6%)	26	—
3^rd^ sampling point	74 (24.1%)	307	7 (25.0%)	28	—
25(OH)D					
1^st^ sampling point	65.5 (16.9)	307	63.0 (17.1)	113	0.19
<50 nmol/L	51 (16.6%)	—	25 (22.1%)	—	—
50–75 nmol/L	167 (54.4%)	—	67 (59.3%)	—	—
>75 nmol/L	89 (29.0%)	—	21 (18.6%)	—	—
2^nd^ sampling point	69.5 (19.1)	307	67.9 (19.8)	121	0.45
<50 nmol/L	47 (15.3%)	—	20 (20.5%)	—	—
50–75 nmol/L	149 (48.5%)	—	62 (16.4%)	—	—
>75 nmol/L	111 (36.2%)	—	40 (32.8%)	—	—
3^rd^ sampling point	73.8 (19.5)	307	71.3 (20.0)	113	0.24
<50 nmol/L	30 (9.8%)	—	14 (12.3%)	—	—
50–75 nmol/L	145 (47.2%)	—	60 (52.6%)	—	—
>75 nmol/L	132 (43.0%)	—	40 (35.1%)	—	—

Abbreviation: SD, standard deviation; 25(OH)D, 25-hydroxyvitamin D; CES-D, Center for Epidemiologic Studies Depression Scale.

**Table 2:** Associations between covariates and depressive symptoms and 25(OH)D levels during pregnancy.

	Depressive symptoms			25(OH)D levels		
	Estimate in SD units	95% CI	*p*	Estimate in SD units	95% CI	*p*
Age <40 vs. ≥40 years	−0.34	−0.68, −0.01	0.05	0.11	−0.72, 0.40	0.43
Upper vs. lower secondary or lower	0.07	−0.13, 0.26	0.51	0.15	−0.02, 0.32	0.08
Smoking during pregnancy						
No	Ref	—	—	Ref	—	—
Quit during the 1^st^ trimester	0.10	−0.42, 0.62	0.70	−0.31	−0.75, 0.15	0.18
Smoked throughout	0.45	−0.40, 1.31	0.30	−0.62	−1.30, 0.06	0.07
Alcohol use during pregnancy vs. no	0.32	0.04, 0.60	0.03	0.17	−0.08, 0.41	0.18
Body mass index in early pregnancy						
<25 kg/m^2^	Ref	—	—	Ref	—	—
25–29.99 kg/m^2^	0.02	−0.23, 0.28	0.86	−0.37	−0.57, −0.18	0.001
⩾30 kg/m^2^	0.25	0.02, 0.47	0.03	−0.44	−0.66, −0.22	0.001
Any hypertensive disorder vs. no disorder	−0.10	−0.31, 0.11	0.34	−0.08	−0.26, 0.10	0.38
Any diabetes disorder vs. no disorder	−0.11	−0.35, 0.13	0.35	−0.09	−0.30, 0.11	0.37

Associations measured with linear mixed-model regression analysis with depressive symptoms and 25(OH)D levels treated as time-varying within-person outcome and covariates treated as a time-varying within-person predictor. Gestational week at blood sampling was included as a time-varying within-person predictor. Estimates and 95% CIs reflect differences between categorical covariate variable groups in SD units. SD, standard deviation; CI, confidence interval, and 25(OH)D, 25-hydroxyvitamin D.

**Table 3:** Linear regression associations of 25(OH)D and depressive symptoms separately at the three measurement points during pregnancy.

Depressive symptoms (outcome)	*B* (95% CI)	*p*
25(OH)D (predictor) at 1^st^ sampling point		
CES-D at 11.6–15.1 weeks	0.01 (−0.12, 0.10)	0.85
CES-D at 17.1–22.9 weeks	0.09 (−0.02, 0.20)	0.12
CES-D at 25.3–30.6 weeks	0.11 (−0.00, 0.22)	0.051
25(OH)D at 2^nd^ sampling point		
CES-D at 17.1–22.9 weeks	−0.03 (−0.15, 0.08)	0.58
CES-D at 25.3–30.6 weeks	0.05 (−0.06, 0.16)	0.38
25(OH)D at 3^rd^ sampling point		
CES-D at 25.3–30.6 weeks	0.01 (−0.11, 0.12)	0.91

*B* and 95% CI from linear regression analyses refer to change in SD units in depressive symptoms per SD unit change in 25(OH)D. CI, confidence interval; 25(OH)D, 25-hydroxyvitamin D; CES-D, Center for Epidemiologic Studies Depression Scale.

**Table 4:** Logistic regression associations of clinically relevant depressive symptoms (≥16 points in CES-D) and vitamin D deficiency (25(OH)D <50 nmol/L) separately at the three measurement points during pregnancy.

Clinically relevant depressive symptoms, CES-D ≥16 points (outcome)	Exp (*B*) (95% CI)	*p*
25(OH)D (predictor) at 1^st^ sampling point		
CES-D at 11.6–15.1 weeks	1.75 (0.90; 3.40)	0.10
CES-D at 17.1–22.9 weeks	0.51 (0.22; 1.19)	0.12
CES-D at 25.3–30.6 weeks	0.84 (0.41; 1.74)	0.64
25(OH)D at 2^nd^ sampling point		
CES-D at 17.1–22.9 weeks	0.81 (0.37; 1.76)	0.59
CES-D at 25.3–30.6 weeks	0.83 (0.39; 1.76)	0.62
25(OH)D at 3^rd^ sampling point		
CES-D at 25.3–30.6 weeks	1.16 (0.49; 2.73)	0.73

Exp (*B*) and 95% CI from logistic regression analysis refer to the association between dichotomized depressive symptoms (clinically significant levels of depressive symptoms; CES-D ≥ 16 points) and 25(OH)D variable (vitamin D deficiency; 25(OH)D <50 nmol/L. CI, confidence interval; 25(OH)D, 25-hydroxyvitamin D; and CES-D, Center for Epidemiologic Studies Depression Scale.

**Table 5:** Associations of 25(OH)D and depressive symptoms across the three measurement points during pregnancy.

Depressive symptoms during pregnancy, continuous score (outcome)		Estimate in SD units (95% CI)	*p*
Association between levels of 25(OH)D and depressive symptoms	Model 1	−0.05 (−0.12, 0.01)	0.12
	Model 2	−0.05 (−0.11, 0.02)	0.14

Association between the increase in 25(OH)D and depressive symptoms	Model 1	−1.41 (−2.75, −0.07)	0.039
	Model 2	−1.20 (−2.54, 0.14)	0.079

Model 1 unadjusted for covariates but includes the gestational week when the blood was sampled as a within-person time-varying predictor. Model 2 is Model 1 + season when the blood was sampled, age, alcohol use, body mass index in early pregnancy (categorized as normal weight (<25 kg/m^2^), overweight (25–29.99 kg/m^2^), and obese (⩾30 kg/m^2^)). Estimates and 95% confidence intervals (95% CI) reflect a change in SD units in depressive symptoms per SD unit change in 25(OH)D during pregnancy derived from linear mixed-model regression analyses.

**Table 6:** Associations of 25(OH)D and depressive symptoms across the three measurement points during pregnancy among women without a history of depression diagnoses before pregnancy (*n* = 270).

Depressive symptoms during pregnancy, continuous score (outcome)		Estimate in SD units (95% CI)	*p*
Association between levels of 25(OH)D and depressive symptoms	Model 1	−0.05 (−0.12, 0.02)	0.16
	Model 2	−0.05 (−0.12, 0.02)	0.14

Association between the change of 25(OH)D and depressive symptoms	Model 1	−1.56 (−2.91, −0.21)	0.024
	Model 2	−1.19 (−2.54, 0.16)	0.084

Model 1 unadjusted for covariates but includes the gestational week when the blood was sampled as a within-person time-varying predictor. Model 2 is Model 1 + season when the blood was sampled, age, alcohol use, body mass index in early pregnancy (categorized as normal weight (<25 kg/m^2^), overweight (25–29.99 kg/m^2^), and obese (⩾30 kg/m^2^)). Estimates and 95% confidence intervals (95% CI) reflect a change in SD units in depressive symptoms per SD unit change in 25(OH)D during pregnancy derived from linear mixed-model regression analyses.

## Data Availability

The data that support the findings of this study are available on request from the corresponding author. The data are not publicly available due to privacy or ethical restrictions. Data requests may be subject to further review by the Finnish national register authorities and by the ethical committees.

## References

[1] Lahti M., Savolainen K., Tuovinen S. (2017). Maternal depressive symptoms during and after pregnancy and psychiatric problems in children. *Journal of the American Academy of Child & Adolescent Psychiatry*.

[2] Molyneaux E., Poston L., Ashurst-Williams S., Howard L. M. (2014). Obesity and mental disorders during pregnancy and postpartum: a systematic review and meta-analysis. *Obstetrics & Gynecology*.

[3] Tuovinen S., Lahti-Pulkkinen M., Girchenko P. (2018). Maternal depressive symptoms during and after pregnancy and child developmental milestones. *Depression and Anxiety*.

[4] Vesga-López O., Blanco C., Keyes K., Olfson M., Grant B. F., Hasin D. S. (2008). Psychiatric disorders in pregnant and postpartum women in the United States. *Archives of General Psychiatry*.

[5] Leigh B., Milgrom J. (2008). Risk factors for antenatal depression, postnatal depression and parenting stress. *BMC Psychiatry*.

[6] Heron J., O’Connor T. G., Evans J., Golding J., Glover V. (2004). The course of anxiety and depression through pregnancy and the postpartum in a community sample. *Journal of Affective Disorders*.

[7] Jarde A., Morais M., Kingston D. (2016). Neonatal outcomes in women with untreated antenatal depression compared with women without depression: a systematic review and meta-analysis. *JAMA Psychiatry*.

[8] Grigoriadis S., VonderPorten E. H., Mamisashvili L. (2013). The impact of maternal depression during pregnancy on perinatal outcomes: a systematic review and meta-analysis. *The Journal of Clinical Psychiatry*.

[9] Pesonen A.-K., Lahti M., Kuusinen T. (2016). Maternal prenatal positive affect, depressive and anxiety symptoms and birth outcomes: The PREDO study. *PLOS ONE*.

[10] Tuovinen S., Lahti-Pulkkinen M., Rantalainen V., Kajantie E., Räikkönen K. (2020). Prenatal programming of child neurocognitive abilities and maternal mental health. *Current Opinion in Endocrine and Metabolic Research*.

[11] Tuovinen S., Lahti-Pulkkinen M., Girchenko P. (2021). Maternal antenatal stress and mental and behavioral disorders in their children. *Journal of Affective Disorders*.

[12] Henry H. L., Bouillon R., Norman A. W. (2010). 14th vitamin D workshop consensus on vitamin D nutritional guidelines. *The Journal of Steroid Biochemistry and Molecular Biology*.

[13] Eyles D. W., Feron F., Cui X. (2009). Developmental vitamin D deficiency causes abnormal brain development. *Psychoneuroendocrinology*.

[14] Harms L. R., Burne T. H. J., Eyles D. W., McGrath J. J. (2011). Vitamin D and the brain. *Best Practice & Research Clinical Endocrinology & Metabolism*.

[15] Kesby J. P., Eyles D. W., Burne T. H. J., McGrath J. J. (2011). The effects of vitamin D on brain development and adult brain function. *Molecular and Cellular Endocrinology*.

[16] Fernandes de Abreu D. A., Eyles D., Féron F. (2009). Vitamin D, a neuro-immunomodulator: Implications for neurodegenerative and autoimmune diseases. *Psychoneuroendocrinology*.

[17] Cannell J. J., Grant W. B., Holick M. F. (2014). Vitamin D and inflammation. *Dermato-Endocrinology*.

[18] Lahti-Pulkkinen M., Girchenko P., Robinson R. (2020). Maternal depression and inflammation during pregnancy. *Psychological Medicine*.

[19] Wagner C. L., Hollis B. W. (2022). The extraordinary metabolism of vitamin D. *eLife*.

[20] Best C. M., Pressman E. K., Queenan R. A., Cooper E., O’Brien K. O. (2019). Longitudinal changes in serum vitamin D binding protein and free 25-hydroxyvitamin D in a multiracial cohort of pregnant adolescents. *The Journal of Steroid Biochemistry and Molecular Biology*.

[21] Jones K. S., Meadows S. R., Schoenmakers I., Prentice A., Moore S. E. (2020). Vitamin D status increases during pregnancy and in response to vitamin D supplementation in rural gambian women. *The Journal of Nutrition*.

[22] Ashley B., Simner C., Manousopoulou A. (2022). Placental uptake and metabolism of 25(OH) vitamin D determine its activity within the fetoplacental unit. *eLife*.

[23] Murphy P. K., Wagner C. L. (2008). Vitamin D and mood disorders among women: an integrative review. *Journal of Midwifery & Women’s Health*.

[24] Aghajafari F., Letourneau N., Mahinpey N., Cosic N., Giesbrecht G. (2018). Vitamin D deficiency and antenatal and postpartum depression: a systematic review. *Nutrients*.

[25] Fallah M., Askari G., Asemi Z. (2020). Is vitamin D status associated with depression, anxiety and sleep quality in pregnancy: a systematic review. *Advanced Biomedical Research*.

[26] Trujillo J., Vieira M. C., Lepsch J. (2018). A systematic review of the associations between maternal nutritional biomarkers and depression and/or anxiety during pregnancy and postpartum. *Journal of Affective Disorders*.

[27] Brandenbarg J., Vrijkotte T. G. M., Goedhart G., van Eijsden M. (2012). Maternal early-pregnancy vitamin D status is associated with maternal depressive symptoms in the amsterdam born children and their development cohort. *Psychosomatic Medicine*.

[28] Gould J. F., Gibson R. A., Green T. J., Makrides M. (2022). A systematic review of vitamin D during pregnancy and postnatally and symptoms of depression in the antenatal and postpartum period from randomized controlled trials and observational studies. *Nutrients*.

[29] Huang J. Y., Arnold D., Qiu C.-F., Miller R. S., Williams M. A., Enquobahrie D. A. (2014). Association of serum vitamin D with symptoms of depression and anxiety in early pregnancy. *Journal of Women’s Health*.

[30] Cassidy-Bushrow A. E., Peters R. M., Johnson D. A., Li J., Rao D. S. (2012). Vitamin D nutritional status and antenatal depressive symptoms in African American women. *Journal of Women’s Health*.

[31] Jani R., Knight-Agarwal C. R., Bloom M., Takito M. Y. (2020). The association between pre-pregnancy body mass index, perinatal depression and maternal vitamin D status: findings from an Australian cohort study. *International Journal of Women’s Health*.

[32] Lamb A. R., Lutenbacher M., Wallston K. A., Pepkowitz S. H., Holmquist B., Hobel C. J. (2018). Vitamin D deficiency and depressive symptoms in the perinatal period. *Archives of Women’s Mental Health*.

[33] Cunha Figueiredo A. C., Trujillo J., Freitas-Vilela A. A. (2017). Association between plasma concentrations of vitamin D metabolites and depressive symptoms throughout pregnancy in a prospective cohort of Brazilian women. *Journal of Psychiatric Research*.

[34] Williams J. A., Romero V. C., Clinton C. M. (2016). Vitamin D levels and perinatal depressive symptoms in women at risk: a secondary analysis of the mothers, omega-3, and mental health study. *BMC Pregnancy and Childbirth*.

[35] Vaziri F., Nasiri S., Tavana Z., Dabbaghmanesh M. H., Sharif F., Jafari P. (2016). A randomized controlled trial of vitamin D supplementation on perinatal depression: in Iranian pregnant mothers. *BMC Pregnancy and Childbirth*.

[36] Girchenko P., Hämäläinen E., Kajantie E. (2016). Prediction and prevention of preeclampsia and intrauterine growth restriction (PREDO) study. *International Journal of Epidemiology*.

[37] von Elm E., Altman D. G., Egger M., Pocock S. J., Gøtzsche P. C., Vandenbroucke J. P. (2008). The strengthening the reporting of observational studies in epidemiology (STROBE) statement: guidelines for reporting observational studies. *Journal of Clinical Epidemiology*.

[38] Radloff L. S. (1977). The CES-D scale. *Applied Psychological Measurement*.

[39] Heller H. M., Draisma S., Honig A. (2022). Construct validity and responsiveness of instruments measuring depression and anxiety in pregnancy: a comparison of EPDS, HADS-A and CES-D. *International Journal of Environmental Research and Public Health*.

[40] Maloni J. A., Park S., Anthony M. K., Musil C. M. (2005). Measurement of antepartum depressive symptoms during high-risk pregnancy. *Research in Nursing & Health*.

[41] Natamba B. K., Achan J., Arbach A. (2014). Reliability and validity of the center for epidemiologic studies-depression scale in screening for depression among HIV-infected and -uninfected pregnant women attending antenatal services in northern Uganda: a cross-sectional study. *BMC Psychiatry*.

[42] Rosecrans R., Dohnal J. C. (2014). Seasonal vitamin D changes and the impact on health risk assessment. *Clinical Biochemistry*.

[43] Aasheim V., Waldenström U., Hjelmstedt A., Rasmussen S., Pettersson H., Schytt E. (2012). Associations between advanced maternal age and psychological distress in primiparous women, from early pregnancy to 18 months postpartum. *BJOG: An International Journal of Obstetrics & Gynaecology*.

[44] Brot C., Rye Jørgensen N., Sørensen O. H. (1999). The influence of smoking on vitamin D status and calcium metabolism. *European Journal of Clinical Nutrition*.

[45] Luger T. M., Suls J., Vander Weg M. W. (2014). How robust is the association between smoking and depression in adults? A meta-analysis using linear mixed-effects models. *Addictive behaviors*.

[46] Walsh J. S., Bowles S., Evans A. L. (2017). Vitamin D in obesity. *Current Opinion in Endocrinology, Diabetes & Obesity*.

[47] Shay M., MacKinnon A. L., Metcalfe A. (2020). Depressed mood and anxiety as risk factors for hypertensive disorders of pregnancy: a systematic review and meta-analysis. *Psychological Medicine*.

[48] O’Callaghan K. M., Kiely M. (2018). Systematic review of vitamin D and hypertensive disorders of pregnancy. *Nutrients*.

[49] Pleskačová A., Bartáková V., Pácal L. (2015). Vitamin D status in women with gestational diabetes mellitus during pregnancy and postpartum. *BioMed Research International*.

[50] Lee K. W., Ching S. M., Devaraj N. K. (2020). Diabetes in pregnancy and risk of antepartum depression: a systematic review and meta-analysis of cohort studies. *International Journal of Environmental Research and Public Health*.

[51] Carlson C. R., Uriu-Adams J. Y., Chambers C. D. (2017). Vitamin D deficiency in pregnant ukrainian women: effects of alcohol consumption on vitamin D status. *Journal of the American College of Nutrition*.

[52] Bolton H. L., Hughes P. M., Turton P., Sedgwick P. (1998). Incidence and demographic correlates of depressive symptoms during pregnancy in an inner London population. *Journal of Psychosomatic Obstetrics & Gynecology*.

[53] Lewinsohn P. M., Seeley J. R., Roberts R. E., Allen N. B. (1997). Center for epidemiologic studies depression scale (CES-D) as a screening instrument for depression among community-residing older adults.. *Psychology and Aging*.

[54] Wang J., Liu N., Sun W., Chen D., Zhao J., Zhang W. (2018). Association between vitamin D deficiency and antepartum and postpartum depression: a systematic review and meta-analysis of longitudinal studies. *Archives of Gynecology and Obstetrics*.

[55] Marcus S. M., Flynn H. A., Blow F. C., Barry K. L. (2003). Depressive symptoms among pregnant women screened in obstetrics settings. *Journal of Women’s Health*.

[56] Vellekkatt F., Menon V. (2019). Efficacy of vitamin D supplementation in major depression: a meta-analysis of randomized controlled trials. *Journal of Postgraduate Medicine*.

[57] Omidvar S., Faramarzi M., Hajian-Tilak K., Nasiri Amiri F., Rohrmann S. (2018). Associations of psychosocial factors with pregnancy healthy life styles. *PLOS ONE*.

[58] van Lee L., Chia A., Phua D. (2020). Multiple modifiable lifestyle factors and the risk of perinatal depression during pregnancy: findings from the GUSTO cohort. *Comprehensive Psychiatry*.

[59] Jääskeläinen T., Knekt P., Marniemi J. (2013). Vitamin D status is associated with sociodemographic factors, lifestyle and metabolic health. *European Journal of Nutrition*.

[60] Hahn-Holbrook J., Davis E. P., Sandman C. A., Glynn L. M. (2023). Maternal prenatal cortisol trajectories predict accelerated growth in infancy. *Psychoneuroendocrinology*.

[61] Raulio S., Erlund I., Männistö S. (2017). Successful nutrition policy: improvement of vitamin D intake and status in Finnish adults over the last decade. *The European Journal of Public Health*.

[62] Pallasmaa N., Ekblad U., Gissler M., Alanen A. (2015). The impact of maternal obesity, age, pre-eclampsia and insulin dependent diabetes on severe maternal morbidity by mode of delivery—a register-based cohort study. *Archives of Gynecology and Obstetrics*.

[63] Lindeman S., Hämäläinen J., Isometsä E. (2000). The 12-month prevalence and risk factors for major depressive episode in Finland: representative sample of 5993 adults. *Acta Psychiatrica Scandinavica*.

[64] Markkula N., Suvisaari J., Saarni S. I. (2015). Prevalence and correlates of major depressive disorder and dysthymia in an eleven-year follow-up-results from the Finnish Health 2011 survey. *Journal of Affective Disorders*.

[65] American Psychiatric Association (2013). *Diagnostic and Statistical Manual of Mental Disorders*.

[66] Finnish Food Authority (2014). Special instructions and restrictions;. https://www.ruokavirasto.fi/en/foodstuffs/healthy-diet/nutrition-and-food-recommendations/special-instructions-and-restrictions/.

[67] Cashman K. D., van den Heuvel E. G. H. M., Schoemaker R. J. W., Prévéraud D. P., Macdonald H. M., Arcot J. (2017). 25-hydroxyvitamin D as a biomarker of vitamin D status and its modeling to inform strategies for prevention of vitamin D deficiency within the population. *Advances in Nutrition*.

